# A randomized control trial to evaluate the importance of pre-training basic laparoscopic psychomotor skills upon the learning curve of laparoscopic intra-corporeal knot tying

**DOI:** 10.1186/s10397-017-1031-3

**Published:** 2017-12-20

**Authors:** Carlos Roger Molinas, Maria Mercedes Binda, Cesar Manuel Sisa, Rudi Campo

**Affiliations:** 1 NEOLIFE—Medicina y Cirugía Reproductiva, Avenida Brasilia 760, 1434 Asunción, Paraguay; 2 Faculty of Medicine, Universidad del Pacífico Privada, Avenida San Martín 961, 1813 Asunción, Paraguay; 3 The European Academy of Gynaecological Surgery, Diestsevest 43, bus 0001, B3000 Leuven, Belgium

**Keywords:** Laparoscopy, Training, Intra-corporeal knot tying, Psychomotor skills, Education, Training box, ENCILAP model, LASTT model

## Abstract

**Background:**

Training of basic laparoscopic psychomotor skills improves the acquisition of more advanced laparoscopic tasks, such as laparoscopic intra-corporeal knot tying (LICK). This randomized controlled trial was designed to evaluate whether pre-training of basic skills, as laparoscopic camera navigation (LCN), hand-eye coordination (HEC), and bimanual coordination (BMC), and the combination of the three of them, has any beneficial effect upon the learning curve of LICK. The study was carried out in a private center in Asunción, Paraguay, by 80 medical students without any experience in surgery. Four laparoscopic tasks were performed in the ENCILAP model (LCN, HEC, BMC, and LICK). Participants were allocated to 5 groups (G1–G5). The study was structured in 5 phases. In phase 1, they underwent a base-line test (*T*_1_) for all tasks (1 repetition of each task in consecutive order). In phase 2, participants underwent different training programs (30 consecutive repetitions) for basic tasks according to the group they belong to (G1: none; G2: LCN; G3: HEC; G4: BMC; and G5: LCN, HEC, and BMC). In phase 3, they were tested again (*T*_2_) in the same manner than at *T*_1_. In phase 4, they underwent a standardized training program for LICK (30 consecutive repetitions). In phase 5, they were tested again (*T*_3_) in the same manner than at *T*_1_ and *T*_2_. At each repetition, scoring was based on the time taken for task completion system.

**Results:**

The scores were plotted and non-linear regression models were used to fit the learning curves to one- and two-phase exponential decay models for each participant (individual curves) and for each group (group curves). The LICK group learning curves fitted better to the two-phase exponential decay model. From these curves, the starting points (*Y*0), the point after HEC training/before LICK training (*Y*1), the Plateau, and the rate constants (*K*) were calculated. All groups, except for G4, started from a similar point (*Y*0). At *Y*1, G5 scored already better than the others (G1 *p* = .004; G2 *p* = .04; G3 *p* < .0001; G4 NS). Although all groups reached a similar Plateau, G5 has a quicker learning than the others, demonstrated by a higher *K* (G1 *p* < 0.0001; G2 *p* < 0.0001; G3 *p* < 0.0001; and G4 *p* < 0.0001).

**Conclusions:**

Our data confirms that training improves laparoscopic skills and demonstrates that pre-training of all basic skills (i.e., LCN, HEC, and BMC) shortens the LICK learning curve.

**Electronic supplementary material:**

The online version of this article (10.1186/s10397-017-1031-3) contains supplementary material, which is available to authorized users.

## Background

The ideal method for training in laparoscopic surgery is continuously debated, and several systems have been proposed and developed based upon different models, target population, local institutional characteristics, medical specialty, and others [1, 2].

In spite of the controversies and differences, most specialists in surgical education agree that the traditional apprentice-tutor model is no longer useful for training all skills necessary for laparoscopic surgery. Indeed, to achieve proficiency through this model seems unacceptable for both practical and ethical reasons, such as the limited number of tutors, the fewer surgical cases in daily practice, the increased operating time, the higher complication rate, and the long learning curves [3, 4].

Furthermore, laparoscopic surgery demands both surgical and psychomotor skills that not necessarily should be trained together [3, 4], with increasing evidence suggesting that psychomotor skills must be trained earlier and outside the operating room [5–10].

Following this philosophy, the European Academy of Gynecological Surgery has developed the LASTT (Laparoscopic Skills Training and Testing) model for training basic laparoscopic psychomotor skills, such as laparoscopic camera navigation (LCN), hand-eye coordination (HEC), and bimanual coordination (BMC). The feasibility, face validity, and construct validity of this model have been demonstrated [11–13], and together with other tools (i.e., SUTT model, HYSTT model, E-Knot model, The Winner Project [14]), the LASTT model is also currently used for certification purposes [3, 4, 15, 16].

In the LASTT model, it has also been confirmed that training improves the laparoscopic skills, and it has been demonstrated that HEC training with both hands (dominant hand and non-dominant hand) improves the acquisition and retention of more complex laparoscopic tasks, such as intra-corporeal knot tying (LICK) [12, 17], shortening the LICK learning curve [18]. Therefore, this study was designed to evaluate whether pre-training of basic skills, as LCN, HEC or BMC, and the combination of the three of them, has any beneficial effect upon the learning curve of LICK.

## Methods

### Participants and venue

The study was approved by the Institutional Review Board and performed at *Universidad del Pacífico Privada* in Asunción, Paraguay, in 2012 by 80 last-year medical students without any experience in surgery. The sample size was calculated based on the LICK scores. Taking into account base-line scores of 500 ± 250 (mean ± SD) reported in novices [12] and to be able to detect a 50%difference with a power of 80% and a two-tailed level of significance of .05, some 12 participants per group would be required.

### Instruments and materials and laparoscopic tasks

**Fig. 1:**
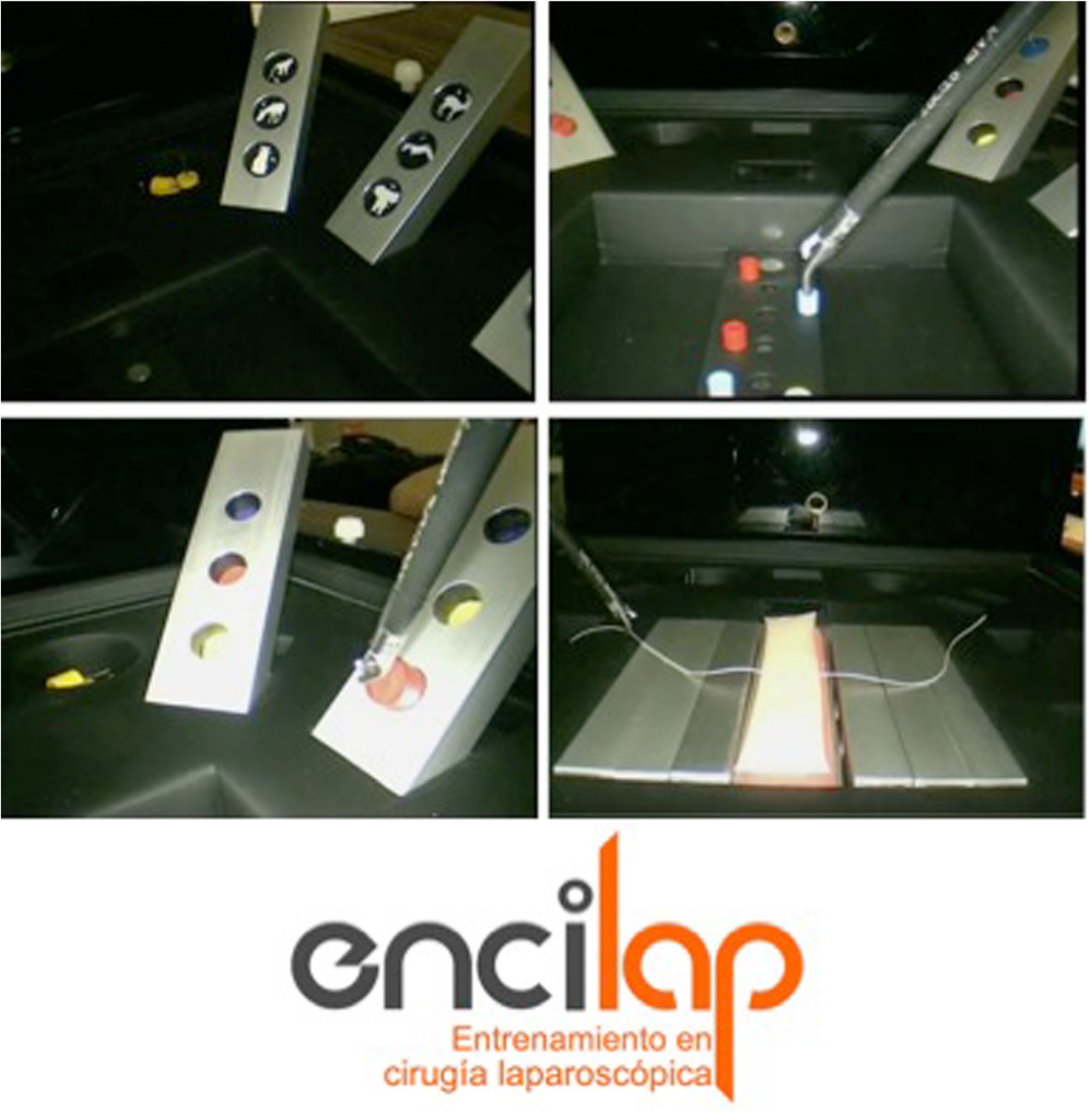
The ENCILAP model

**Fig. 2:**
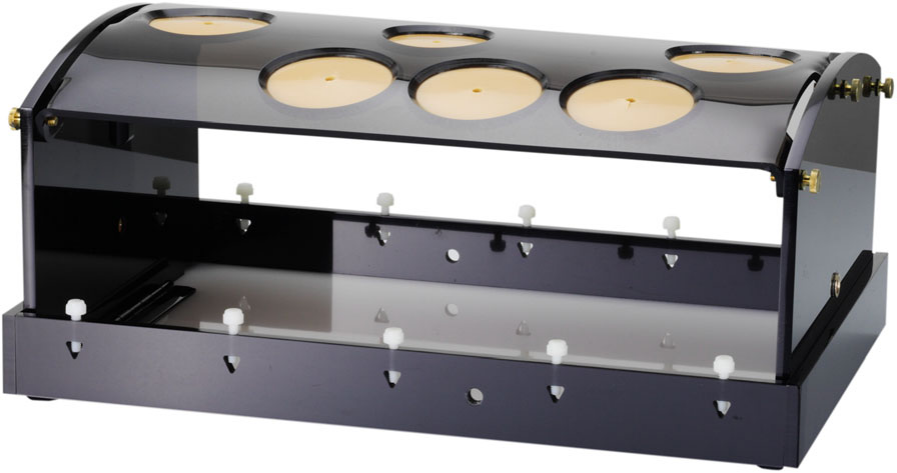
The Szabo trainer box

A novel model (*ENtrenamiento en CIrugía LAParoscópica*: ENCILAP) adapted from the LASTT [13] and the SUTT models was developed (Fig. 1). The model consists in a platform (30 × 30 cm) with several hollows for storing tools and fitting working modules (rectangular blocks of 10 × 2.5 × 1 cm). Some modules are covered with a soft pad, whereas others have three circular wells (1 × 0.5 cm) with a picture or a color bottom. The modules can be fitted at 45°/90° at different locations of the platform. The ENCILAP was placed into the Szabo trainer box (Fig. 2). All tasks were performed with a 10-mm 30° optic connected to a laparoscopic tower and with standard laparoscopic instruments as described for each task (Karl Storz, Tuttlingen, Germany).

For LCN, the ENCILAP model was fitted with 6 modules at 45° (3 at the right and 3 at the left). Participants navigated the camera with the preferred hand in order to identify 12 figures, which comprise a large and a small symbol. The supervisor indicated the first symbol to be shown, which was identifiable from a panoramic viewpoint. Participants looked throughout the model for this symbol, found it, and focused on the small symbol situated next to it, which was shown on the center of the screen from a close-up viewpoint. This small symbol indicated the next large symbol to be identified. Following this order, participants continued till the identification of the last small symbol (Additional file 1: Video 1).

For HEC, the ENCILAP model was fitted with six modules at 45° (three at each side). One well of each module was filled in with a color pushpin with the tail upwards. Six color rings (4 mm with an opening of 2 mm) were placed in the center of the platform. Participants held and navigated the camera with one hand and held a 5-mm Kelly forceps with the other hand. One by one, the rings were grasped and introduced in the tails of the pushpin of the same color (Additional file 2: Video 2). The task was performed and scored alternatively with the dominant hand (DH) and the non-dominant hand (NDH), which was defined by the hand holding the forceps.

For BMC, the ENCILAP model was fitted with six modules at 45° (three at each side). One well of each module at the left was filled in with a color pushpin with the tail upwards. An assistant navigated the camera according to the participant’s instructions. One by one, the pushpins were grasped and lifted with a 5-mm Kelly forceps held with the DH, re-grasped with a similar forceps held with the NDH, and introduced into empty wells of the same color at the right of the model. Then, the pushpins were transferred in the inverse order (from right to left) (Additional file 3: Video 3 and Additional file 4: Video 4).

For LICK, the ENCILAP model was fitted with the soft module at 0° in the center of the platform, which was mounted with a suture (vicryl 2-0, 20 cm length) with 1 cm between entry and exit sites and with tails equally distributed to both sides. An assistant navigated the camera according to the participant’s instructions, who held a 5-mm Koh needle holder with the DH and a similar one with the NDH. The tip of the thread was grasped with the left needle holder and the thread was pulled through the pad, leaving a 2-cm tail on the opposite side. Then, a double counter-clockwise knot was made, followed by a single clockwise knot, and, finally, by a single counter-clockwise knot (Additional file 5: Video 5). The time for each repetition was limited to 300 s. The repetition ended either when the knot was accomplished or when the time limit expired. The supervisor controlled the knot quality and only flat and square knots were considered correctly performed.

### Scoring system

The scoring was based in the time taken for task completion system. Each repetition of each task was scored by a supervisor, as explained below. For LCN, HEC, and BMC, the time for each repetition was limited to 180 s. The repetition ended either when all objectives (12 figures identified for LCN, 6 rings transported for HEC, 6 pushpins transported for BMC) were accomplished or when the time limit expired. The quality of the objectives was obvious, and thus, the supervisor just counted the number of objectives accomplished. If within the time limit at least one objective was achieved, the score was calculated dividing the time actually used (1–180) by the number of objectives accomplished (LCN 1–12, HEC 1–6, BMC 1–6). If in the maximum time any objective was achieved, a penalty score of 360 was given.

For LICK, the time for each repetition was limited to 300 s. The repetition ended either when the knot was accomplished or when the time limit expired. Since the quality of the knot can be debatable, the supervisor controlled the quality and only flat and square knots were considered correct. If within the time limit the knot was successfully executed, the score was the time actually used (1–300). If in the maximum time the knot was not successfully executed, a penalty score of 600 was given.

### Experimental design

**Fig. 3:**
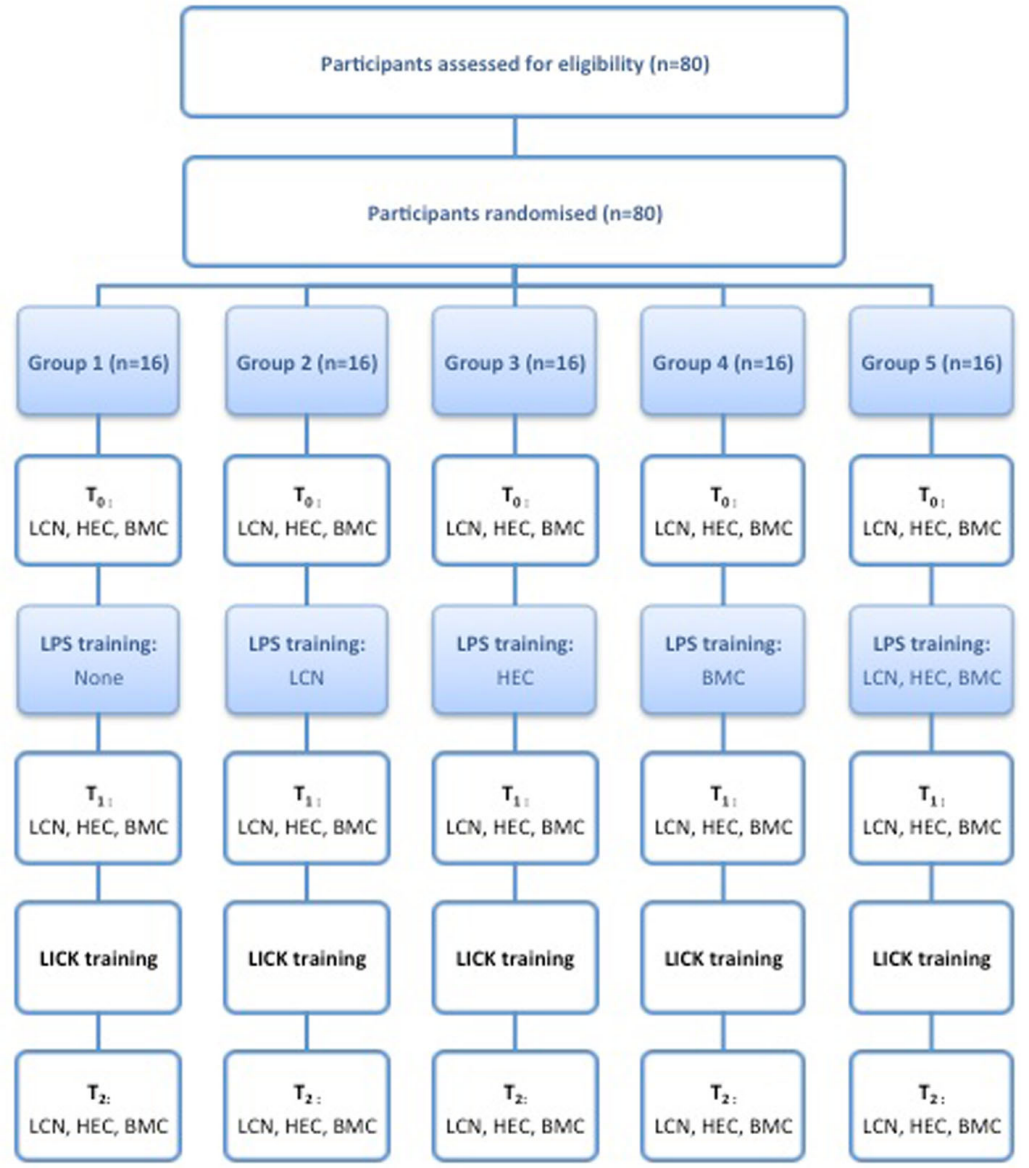
Flow chart. LPS laparoscopic psychomotor skills, LCN laparoscopic camera navigation, HEC hand-eye coordination, BMC bimanual coordination, LICK laparoscopic intra-corporeal knot tying

Participants were randomly allocated to five groups (G1, G2, G3, G4, or G5; *n* = 16 per group). Within each group, they worked in fixed pairs throughout the study. Working sessions of 1–3 h were performed 2–3 times a week. A supervisor was present at the working station in all sessions to ascertain the setup was correctly ensembled and to score the tasks. The study was structured in five phases (Fig. 3).

#### Phase 1

In the first session, participants filled in a questionnaire reporting age, gender, dominant hand side, hands’ size according to the gloves’ size (small 6.0–6.5, medium 7.0–7.5, large 8.0–8.5), interest in surgery, and experience in video games according to a visual analogue scale from 0 (no) to 10 (a lot). Then, video demonstration and full explanation of the different tasks were provided only one time. Finally, participants performed a baseline test (*T*_1_), consisting in one repetition of LCN, HEC, BMC, and LICK in consecutive order and which were scored by the supervisor, as explained above.

#### Phase 2

Participants underwent differentiated training for basic skills according to the group they belong to, which consisted in 30 consecutive repetitions of the relevant task (G1: none; G2: LCN; G3: HEC; G4: BMC; and G5: LCN, HEC, and BMC in consecutive order). These were done in as many sessions as necessary according to participants’ group and skills, spread in 1–2 weeks for G2, G3, and G4 and in 3–6 weeks for G5. All these repetitions were scored by the supervisor, as explained above.

#### Phase 3

After completion of basic skills training and before LICK training, participants were tested again (*T*_2_) in the same manner than at *T*_1_ in one single session.

#### Phase 4

All participants underwent a standardized training program for LICK, which consisted in 30 consecutive repetitions of the task. This was done in as many sessions as necessary according to participants’ skills, spread in 1–2 weeks. All these repetitions were scored by the supervisor, as explained above.

#### Phase 5

After completion of LICK training, participants were tested again (*T*_3_) in the same manner than at *T*_1_ and *T*_2_ in one single session.

### Statistics and curve fitting

All statistical comparisons were performed with GraphPad Prism 6.00 (GraphPad Software, San Diego, California, USA) and SAS (SAS Institute Inc., Cary, North Carolina, USA). A two-tailed *p* value of < .05 was considered statistically significant.

Intergroup differences in demographic parameters were evaluated with one-way ANOVA with Tukey’s multiple comparison post-test (age, interest in surgery, and experience in video games) and with chi-square test (gender, dominant hand side, and hand size).

The effect of the different pre-training conditions upon the LICK performance was evaluated in two ways: firstly, inter- and intragroup differences in the real scores registered at the evaluation points only (*T*_1_, *T*_2_, and *T*_3_) and secondly, inter- and intragroup differences in the calculated scores at the entire learning curves.

The real scores registered by each group at *T*_1_, *T*_2_, and *T*_3_ (continuous variable) were not normally distributed, and therefore, they are presented as medians (interquartile range). To evaluate intergroup differences at *T*_1_, *T*_2_, and *T*_3_, the Kruskal-Wallis test (with Dunn’s multiple comparison post-test) was used. To evaluate intragroup differences (*T*_1_ vs. *T*_2_, *T*_2_ vs. *T*_3_), the Wilcoxon matched-pairs signed rank test was used.

The real scores registered at all points were plotted to produce the learning curves for each student (individual learning curves) and for each group (group learning curves). Non-linear regression models were used to fit the curves to the one- and two-phase exponential decay models. The one-phase exponential decay model is expressed as *Y* = (*Y*0 − Plateau) × exp. (− *K* × *X*) + Plateau. The two-phase exponential decay model is expressed as *Y* = Plateau + SpanFast × exp. (− *K*Fast × *X*) + SpanSlow × exp. (− *K*Slow × *X*), where SpanFast = (*Y*0 − Plateau) × PercentFast × .01 and SpanSlow = (*Y*0 − Plateau) × (100 − PercentFast) × .01. *Y* is a dependent variable (score) and *X* is an independent variable (number of the repetition of the task). *Y*0 is the *Y* value when *X* is zero (the starting point before any training or *T*_1_). Plateau is the *Y* value at infinite times, expressed in the same units as *Y* (the theoretical best score that can be achieved with infinite practice). *K*, *K*Fast and *K*Slow are rate constants, expressed in reciprocal of the *X* units and which measures the steepness of the curve (higher values of *K* indicates faster learning). Span is the difference between *Y*0 and Plateau, expressed in the same units as *Y* values. PercentFast is the fraction of the Span accounted for by the faster of the two components. For LICK, the *Y*1, which represents the *Y* extrapolated value from *X*1 (the first point of the curve immediately after HEC training/before LICK training or *T*2), was also calculated.

The extra sum-of-squares *F* test was used to evaluate which model fits better (one-phase vs. two-phase exponential decay models) and if one single curve adequately fits for all groups. All curve parameters (continuous variable) were normally distributed and therefore they are presented as means ± SEM. To evaluate intergroup differences at each curve parameter, the one-way ANOVA (with Tukey’s multiple comparison post-test) was used. To evaluate intragroup differences (*Y*0 vs. *Y*1), the paired *t* test was used.

General linear methods (proc GLM) was performed to evaluate simultaneously the effect of independent variables such as age, gender (male/female), dominant hand side (right/left), level of interest in surgery (0–10), level of experience in video games (0–10), hand size (small/medium/large), and study group (G1 to G5) upon the speed of the learning (rate constant *K*), which was obtained for each participant from his/her individual learning curve.

## Results

The demographics of the five groups are presented in Table 1. No intergroup differences were detected for any of the demographic parameters.

**Table 1:** Demographics

	Group				
	G1	G2	G3	G4	G5
Age	24.2 ± 0.7	22.3 ± 0.4	22.0 ± 0.3	22.5 ± 0.4	23.9 ± 1.1
Gender					
Male	7 (44%)	7 (44%)	6 (38%)	5 (31%)	9 (56%)
Female	9 (56%)	9 (56%)	10 (62%)	11 (69%)	7 (44%)
Dominant hand					
Right	13 (81%)	12 (75%)	14 (88%)	16 (100%)	15 (94%)
Left	3 (19%)	4 (25%)	2 (12%)	0 (0%)	1 (6%)
Hands size					
Small	7 (44%)	5 (31%)	7 (44%)	7 (44%)	2 (12%)
Medium	8 (50%)	8 (50%)	6 (38%)	7 (44%)	12 (75%)
Large	1 (6%)	3 (19%)	3 (18%)	2 (12%)	2 (12%)
Interest in surgery	7.4 ± 0.5	7.7 ± 0.6	5.8 ± 0.6	6.6 ± 0.6	7.4 ± 0.7
Experience in video games	3.7 ± 0.9	6.5 ± 0.8	5.1 ± 0.7	4.6 ± 0.7	5.2 ± 1.0

Age, interest in surgery, and experience in video games are presented as mean ± SEM. Gender, dominant hand, and hand size are presented as number (%)

The results of the basic tasks (LCN, HEC, and BMC) were disregarded for the aims of this publication to avoid the presentation of so many data that could make the understanding of LICK data more confusing and unclear. Therefore, only the LICK results are presented here.

The baseline scores before any training (*T*_1_) were similar in all groups (NS). Immediately before LICK training (*T*_2_), the scores decreased in all groups compared to *T*_1_ (G1 *p* = .01; G2 *p* = .01; G3 *p* = .0005; G4 *p* = .0005; G5 *p* < .0001). At this point, G5 scored better than G1 (*p* < .05), G2 (*p* < .05), G3 (NS), and G4 (NS). After LICK training (*T*_3_), the scores further decreased in all groups compared to *T*_2_ (G1 *p* < .0001; G2 *p* < .0001; G3 *p* = .0002; G4 *p* < .0001; G5 *p* < .0001). At this point, G5 scored better than G1 (NS), G2 (NS), G3 (*p* < .05), and G4 (NS) (Table 2).

**Table 2:** Laparoscopic intra-corporeal knot tying scores at the evaluation points (*T*_1_, *T*_2_, and *T*_3_) and learning curve parameters

Score	Groups				
	G1	G2	G3	G4	G5
*T*_1_	600 (600–600)	600 (348–600)	600 (277–600)	600 (238–600)	600 (420 – 600)
*T*_2_	425 (165–600)^a#^	289 (188–600)^a#^	213 (90–525)^a^	163 (82–294)^a^	156 (101 – 186)^a^
*T*_3_	49 (40–57)^a^	55 (48–62)^a^	65 (53–80)^a#^	49 (38–59)^a^	47 (35 – 57)^a^
*Y*0	503.2 ± 23.3^+^	459.9 ± 22.7	467.9 ± 27.1^+^	381.7 ± 21.0	503.0 ± 10.9^+^
*Y*1	416.0 ± 15.1^a#+o^	402.4 ± 16.1^a#+o^	289.5 ± 16.8^a#^	342.3 ± 15.6^#^	196.6 ± 9.2^a^
Plateau	70.3 ± 7.6	70.3 ± 10.3	92.9 ± 5.5	63.7 ± 12.1	63.0 ± 2.0
*K*	0.22 ± 0.02^#o^	0.16 ± 0.02^#o^	0.65 ± 0.09^#^	0.13 ± 0.02^#o^	1.19 ± 0.07

Scores *T*_1_, *T*_2_, and *T*_3_ are presented as medians (interquartile range). Curve parameters (*Y*0, *Y*1, Plateau, and *K*) are presented as means ± SEM

*Intragroup differences (*p* < .05): *T*_1_ vs. *T*_0_, *T*_2_ vs. *T*_1_, and *Y*_1_ vs. *Y*_0_

oIntergroup differences (*p* < .05): vs. G3

+Intergroup differences (*p* < .05): vs. G4

#Intergroup differences (*p* < .05): vs. G5

**Fig. 4:**
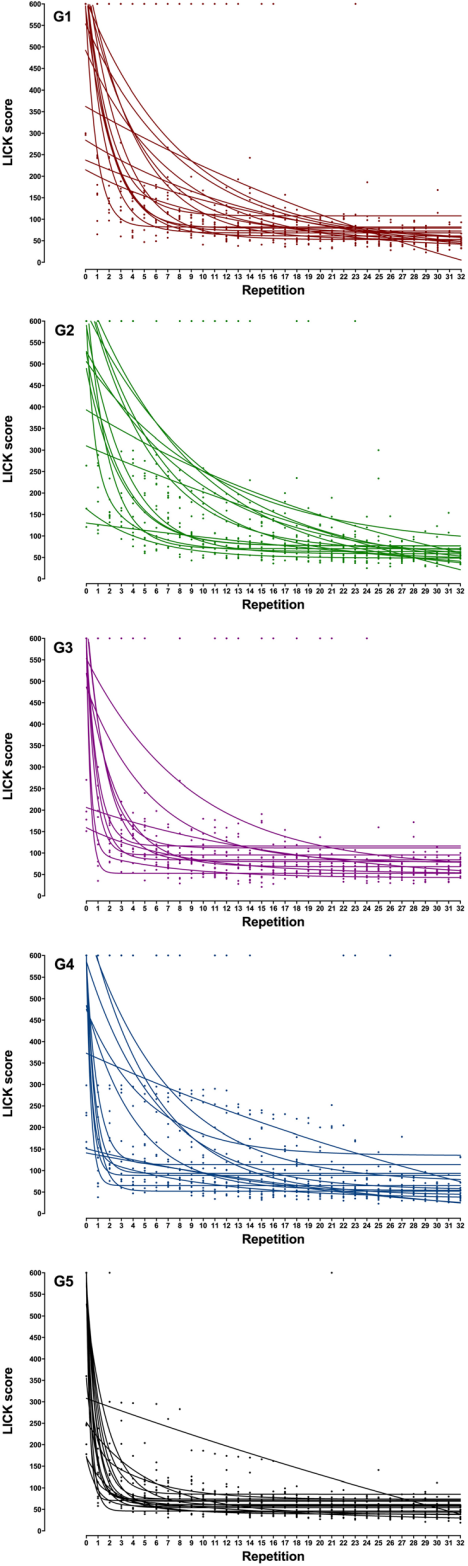
Laparoscopic intra-corporeal knot tying (LICK). Individual learning curves. Each participant performed 33 consecutive repetitions (R0–R32) of LICK. The scores were plotted, and individual learning curves were observed. Most of them fitted to the one-phase exponential decay model, whereas some of them to the two-phase exponential decay model and some did not fit at all to any model

The individual learning curves were fitted to one- and two-phase exponential decay models. Most individual curves fitted better the one-phase model, whereas few of them fitted better to two-phase model or were ambiguous (did not fit to any model) (Fig. 4).

The group learning curves were also fitted to the one- and two-phase exponential decay models. G1 and G2 fitted to both models but fitted better to the two-phase model (G1 *p* = .0005; G2 *p* = .01). G3, G4, and G5 fitted only to the one-phase model. Therefore, the one-phase exponential decay model was used for comparisons. One single type of curve did not adequately fit for all groups (*p* < .0001).

**Fig. 5:**
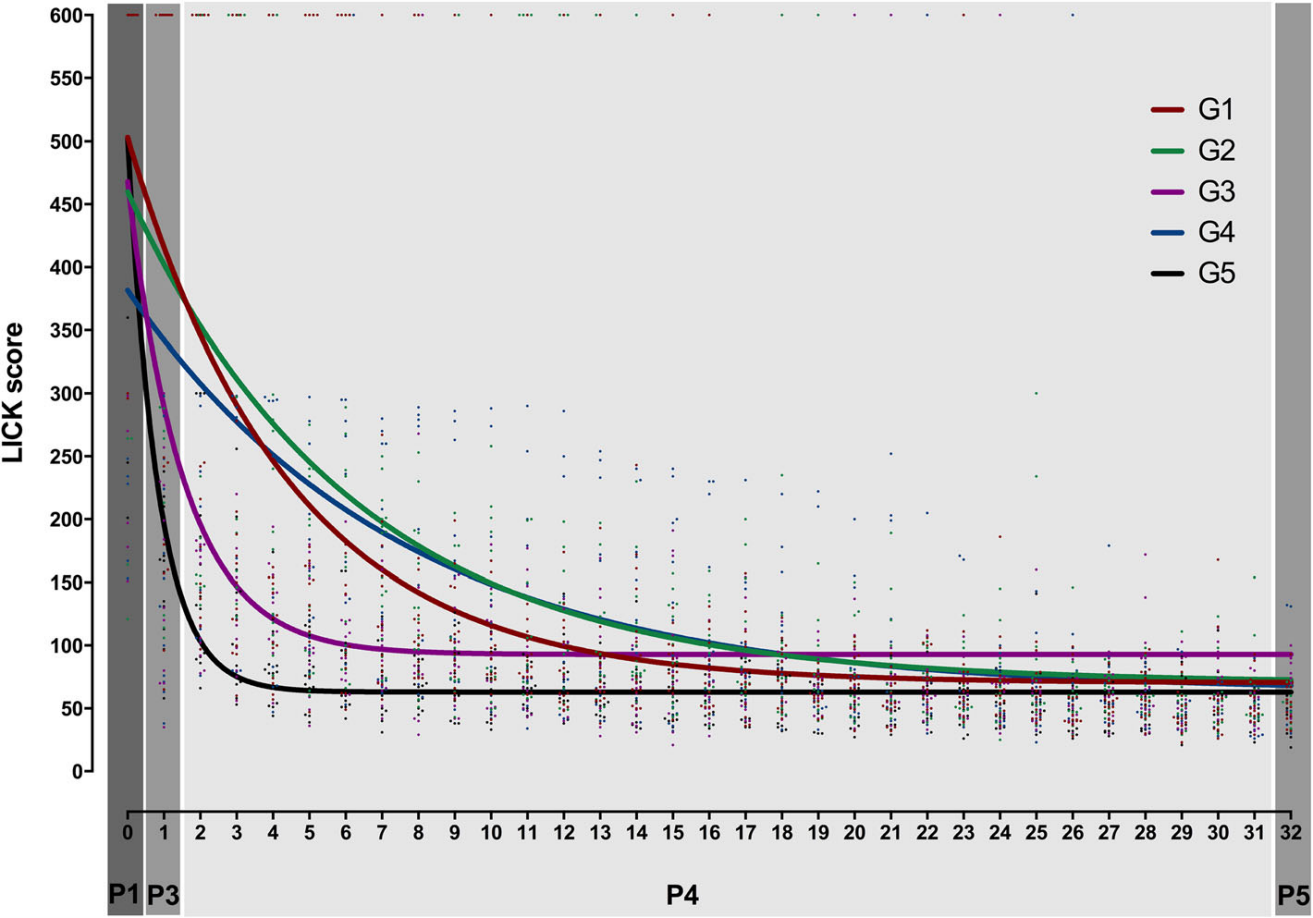
Laparoscopic intra-corporeal knot tying (LICK). Group learning curves. Participants of all groups performed 33 consecutive repetitions (R0–R32) of LICK. Phase 1 (P1): 1 repetition (*T*_1_). Phase 2 (P2): no repetitions (training of basic tasks). Phase 3 (P3): 1 repetition (*T*_2_). Phase 4 (P4): 30 repetitions (LICK training). Phase 5 (P5): 1 repetition (*T*_3_). The scores were plotted, and group learning curves were fitted to a one-phase exponential decay model

All groups had comparable starting points (*Y*0) (NS), except for G4 that started from a lower *Y*0 than G1 (*p* < 0.01), G3 (*p* < 0.05), and G5 (*p* < 0.01). At the next curve value (*Y*1), which represents the value immediately before LICK training, the scores decreased in G1 (*p* = .004), G2 (*p* = .04), G3 (*p* < .0001), G4 (NS), and G5 (*p* < .0001) compared to *Y*0. At this point, G5 scored better than G1 (*p* < .0001), G2 (*p* < .0001), G3 (*p* < .001), and G4 (*p* < .0001), whereas G4 scored better than G1 (*p* < .001) and G2 (*p* < .05), and G3 scored better than G1 (*p* < .0001) and G2 (*p* < .0001). All groups reached a similar plateau (NS) but at different speeds, as demonstrated by the different *K* values (*p* < .0001). Indeed, G5 has a significantly higher *K* than G1 (*p* < 0.0001), G2 (*p* < 0.0001), G3 (*p* < 0.0001), and G4 (*p* < 0.0001), whereas G3 has a significantly higher *K* than G1 (*p* < 0.0001), G2 (*p* < 0.0001), and G4 (*p* < 0.0001) (Table 2 and Fig. 5).

When the effect of the different variables (age, gender, dominant hand side, hand size, interest in surgery, experience in video game, and training group) upon the learning process (evaluated with the learning constant *K*) was computed, only the training group was shown to be significant (*p* = .0005).

## Discussion

This study was performed under the frame of a general project aiming to evaluate factors that potentially can influence laparoscopic skills acquisition, which was initially done in the LASTT model [11–13]. The specific aim of the study was to characterize the LICK learning curve under different pre-training conditions (i.e., one control group with no pre-training, three study groups with pre-training of only one basic skill each (LCN, HEC, or BMC) and one study group with pre-training of the three basic skills (LCN, HEC, and BMC)). This was done in order to define whether pre-training of one basic skill shortens the LICK learning curve and moreover if pre-training of all basic skills has some additive beneficial effect.

For each participant, the scores registered at all points were plotted and individual and group learning curves were observed. For most individuals, as well as for most groups, the one-phase exponential decay was the best-fitted model. From the group curves, the starting point before any training (*Y*0), the point before LICK training (*Y*1), the Plateau, and the learning constant (*K*) were calculated. The *Y*1 was included to evaluate specifically the impact of previous training. Although the real values at baseline of all groups were comparable, the calculated values at *Y*0 were surprisingly different, being better/lower for G4. All other groups had comparable starting points (*Y*0) and improved their scores at the next evaluated point of the curve (i.e., *Y*1). Since this effect was observed even for G1, the influence of repetition cannot be neglected. This effect was more pronounce in G2 and even more in G3 and G4, indicating the importance of training LCN, HEC, and BMC, respectively. However, the greater effect was observed in G5, which also scored significantly better than all other groups. In spite of these differences at the beginning of the curve, all groups reached a similar Plateau but at different speed. Indeed, G5 has a quicker learning as demonstrated by the higher learning constant (*K*). All these together indicate the relevance of training all laparoscopic psychomotor skills first in order not only to start the LICK training from a better point but also to achieve proficiency sooner.

It has been sufficiently proved that training improves laparoscopic skills [19], which also applies specifically to training in box models as recently reported in a meta-analysis, at least in trainees with no previous laparoscopic experience [20]. The majority of the studies base this conclusion upon measurements performed at two or few points (before and after training). The effect of training however can be better appreciated if several points are taken into consideration, allowing tracking the improvement in performance over time, which is defined as a learning curve [21]. Although learning curves have been observed for many health technologies [22], only recently they have become regularly used and reported for laparoscopic procedures [13, 23–27].

Our data about LICK learning curves are consistent with previous studies. Vossen et al. have reported in 29 trainees learning curves with mono- or bi-exponential decay, the latter fitting their experimental points only marginally better [25]. Zhou et al. [28] and Thiyagarajan et al. [29] have also reported in 20 trainees learning curves with an exponential decay shape.

There are few studies evaluating the effect of previous training upon LICK. Consistent with our study, Stefanidis et al. demonstrated in 20 novices that training basic laparoscopic skills (bean drop, running string, block move, checkerboard, and endostitch) shortened the learning curve of a more complex laparoscopic task like suturing. They have also claimed the additional benefit of substantial cost savings because the trained group required significantly less active instruction and less overall costs of the suture material [30]. In spite that learning curves were not reported, Fried et al. have also demonstrated in 215 surgeons that training a basic task (i.e., pegboard transfer) improves significantly the performance of LICK [31].

We believe that one of the strength of our study derives from the study population. Indeed, our sample size was larger than required to achieve our objectives and comprises medical students without experience in practicing surgery (neither laparotomy nor laparoscopy), which ascertains that the skills acquired derive exclusively from the training offered by this study, guaranteeing the purity of the data without external influences. Furthermore, the fact of being last-year students provides them with sufficient knowledge to define their interest in surgical practice. This issue was specifically evaluated, and in spite of the overall great interest reported in the general population, we failed to demonstrate an effect in the results, in contrast with previous studies reporting better results in trainees with higher interest in surgery [27].

We also did not find any effect of the other variables evaluated (age, gender, dominant hand side, hand size, interest in video games), which is consistent with other studies showing that the learning curves are not substantially affected by previous exposure to surgery, either by assisting or by watching laparoscopic interventions, nor by personal characteristics, such as leisure activities, eye dysfunction, eye correction, dominant hand, personality, and gender [25, 27, 31]. For this latter factor, however, Thorson et al. have claimed that among medical students with no previous exposure to laparoscopic trainers, women had a worse performance than men [32], which might be explained by their smaller sample size than in our study (*n* = 32 vs. 80 participants).

As in previous studies [11, 13], our scores were based upon the widely used time taken for task completion system [9, 20, 26, 33]. The system was slightly modified in the sense that the time was limited to 300 s based on previous results indicating that the vast majority of participants would have finished the task within these limits [12]. This was done because it would be practically impossible to carry out a large-scale measurement without time restrictions. It can be argued that this scoring system could be a limitation for our conclusions. We have to admit that time alone is not necessarily an accurate assessment of surgical skills and that accuracy and precision should be incorporated into the scoring system. In our system, however, these factors were implicitly incorporated because only knots correctly performed were scored without penalty.

For the aims of this and future studies, we developed a novel box trainer model, the ENCILAP model, based on the LASTT model [13] in order to make it more versatile and portable and with a more rigorous and precise design. Since the tasks performed were the same as those reported and validated in the LASTT model [11–13] and since this new model is basically the same, except for the design, a specific validation was not necessary. The experience gathered during this and other studies still being conducted, and the data reported, indicates that the simple concept of rectangular block modules placed at different places of a platform, and fitted accordingly, is feasible for training not only the basic skills (i.e., LCN, HEC, BMC) but also LICK, and that can be used as an alternative to the LASTT model or to any other box trainer developed with this aim.

## Conclusions

In conclusion, our study confirms that training improves laparoscopic skills and demonstrates that pre-training of at least one basic task shortens the LICK learning curve and moreover that this beneficial effect is additive and more pronounce when the three basic tasks are pre-trained. Our data also demonstrate that the LICK learning curve is not significantly affected by confounding variables such as age, gender, dominant hand side, hand size, interest in surgery, and interest in video games. The study demonstrates the feasibility of the ENCILAP model for training both basic and advanced laparoscopic skills. It remains to be elucidated the potential effect of continuous tutoring during training and moreover the impact of all these factors upon real surgery in humans.

## Additional files

Additional file 1: Video 1 (MPG 99008 kb)

Additional file 2: Video 2 (MPG 52225 kb)

Additional file 3: Video 3 (MPG 28213 kb)

Additional file 4: Video 4 (MPG 53708 kb)

Additional file 5: Video 5 (MPG 102811 kb)
